# Effectiveness of different tirofiban administration times in patients with no-reflow myocardial infarction during percutaneous coronary intervention

**DOI:** 10.12669/pjms.40.9.10101

**Published:** 2024-10

**Authors:** Chaosheng Mei, Huiping Yu

**Affiliations:** 1 Chaosheng Mei, Department of Cardiovascular Medicine, Hanyang Hospital affiliated to Wuhan University of Science and Technology, Wuhan, Hubei Province 430050, P.R. China; 2 Huiping Yu, Department of Cardiovascular Medicine, Hanyang Hospital affiliated to Wuhan University of Science and Technology, Wuhan, Hubei Province 430050, P.R. China

**Keywords:** Tirofiban, time windows, No-reflow myocardial infarction, Percutaneous coronary intervention

## Abstract

**Objective:**

To compare the effectiveness of different tirofiban administration time windows in patients with no-reflow myocardial infarction (MI) during percutaneous coronary intervention (PCI).

**Methods:**

This single centre retrospective observational study included patients with no-reflow MI, undergoing PCI at the Hanyang Hospital affiliated to Wuhan University of Science and Technology from March 2020 to May 2023. All patients were administered tirofiban. Patients who received tirofiban with postinterventional thrombolysis in myocardial infarction (TIMI) flow ≥ 1 were grouped as Group-I, and patients who were directly given tirofiban through the guiding catheter without forward blood flow were grouped as Group-II. TIMI blood flow classification, levels of cardiac troponin T (cTnT) and creatine kinase isoenzyme MB (CK-MB), incidence of complications and major adverse cardiovascular events (MACE) in the two groups before and after the treatment were statistically analyzed.

**Results:**

A total of 156 patients were included in this study, including 79 patients in Group-I and 77 patients in Group-II. There was no significant difference in the baseline data between the two groups (P>0.05). After treatment, TIMI blood flow classification of the two groups improved and was significantly better in Group-I compared to Group-II (*P*<0.05). After treatment, levels of Serum cTnT and CK-MB in the two groups decreased, and were significantly lower in Group-I than in Group-II (*P*<0.05). There was no significant difference in the incidence of complications between Group-I (3.80%) and Group-II (6.49%) (*P*>0.05). The incidence of MACE in Group-I (3.80%) was lower than that in Group-II (12.99%) (*P*<0.05).

**Conclusions:**

Compared with the direct application of tirofiban, tirofiban given when TIMI Grade≥ 1 for patients with no-reflow MI during PCI can more effectively regulate the blood flow status of target vessels, reduce myocardial injury, and reduce the risk of MACE.

## INTRODUCTION

Myocardial infarction (MI) is one of the life-threatening coronary-associated pathologies characterized by sudden cardiac death with a global prevalence of 3.8%-9.5%.^1^ MI has a great impact on the physical and mental health of patients and imposes a heavy economic burden.^1^-^3^ Percutaneous coronary intervention (PCI) is an important measure for the current clinical treatment of MI, and can effectively restore thrombolysis in myocardial infarction (TIMI) flow.^4^ However, studies have shown that some patients undergoing PCI have diminished myocardial reperfusion, a phenomenon called no-reflow that results from severe microvascular obstruction due to thrombotic events during PCI.^4^,^5^ Therefore, effective anti-platelet aggregation intervention should be taken to inhibit coronary platelet aggregation and thrombosis to avoid no-reflow.^6^

Tirofiban is a commonly used platelet membrane glycoprotein (GP) IIb/IIIa receptor antagonist that can be used in operation and is immediately effective.^7^,^8^ Tirofiban can block the platelet aggregation pathway by competitively inhibiting GP IIb/IIIa receptor, thus preventing platelet aggregation, regulating the blood flow of target vessels during the PCI, and reducing the burden of thrombosis in the lesion.^9^,^10^

However, there is no uniform standard for the optimal administration time of tirofiban in patients with no-reflow myocardial infarction during PCI. Therefore, this study aimed to analyze the clinical data of patients with no-reflow MI undergoing PCI to determine the optimal application time window of tirofiban.

## METHODS

This single centre retrospective observational study included patients with no-reflow MI during PCI treated at the Hanyang Hospital affiliated to Wuhan University of Science and Technology from March 2020 to May 2023. Patients were grouped based on the tirofiban administration time. Patients given tirofiban with TIMI ≥ 1 after the procedure were grouped as Group-I, and patients who were directly given tirofiban through the guiding catheter without forward blood flow were grouped as Group-II.

### Inclusion & Exclusion Criteria:

Patients were included if the patients diagnosed with MI,^11^ had PCI indications and received PCI treatment, no-reflow occurred during the operation, no thrombolytic therapy was given before PCI, and the clinical data were complete. Patients were excluded if no forward blood flow was due to target vasospasm/dissection, with history of tirofiban use before inclusion in the study, treated with the suction catheter during the operation, with multiple organ dysfunction, with severe uncontrolled hypertension (blood pressure ≥ 180/110mmhg); or with hematological diseases.

All procedures performed in study involving human participants were in accordance with the ethical standards of the institutional and/or national research committee(s) and with the Helsinki Declaration (as revised in 2013). The informed consent was waived by the ethics committee for the observational and retrospective nature.

### Ethical Approval:

The ethics committee of our hospital approved this study (No. WHKY2023110101, Date: November 1^st^ 2023).

### Therapeutic method:

Aspirin (manufacturer: Quimica Farmaceutica Bayer S.A.; Germany) 300 mg and clopidogrel (manufacturer: Sanofi Pharmaceutical Co., Ltd; France) 300 mg were taken orally before the operation, and 100 U/kg unfractionated heparin (manufacturer: Jiangsu Wanbang Biochemical Pharmaceutical Group Co., Ltd; China) was given during the operation. 1000 U unfractionated heparin was added every 60 minutes. Group-I was given tirofiban (manufacturer: Lunanbet Pharmaceutical Co., Ltd; China) with loading dose when TIMI Grade-≥ 1 after balloon and guide wire pre-expansion. In Group-II, tirofiban was administered directly through the guiding catheter without forward blood flow. Both groups were given a loading dose of 10 UG/kg, the bolus injection was completed within three minutes, and the pump was maintained at 0.15 ug/(kg·minute) for 36 hours. All patients were given 4000 U enoxaparin (manufacturer: Hubei Yinuorui Biopharmaceutical Co., Ltd; China) for three days.

### Outcome measures:

- TIMI blood flow classification^12^ No forward blood flow at the distal end of the target vessel was classified as Grade-0. Forward blood flow in the distal main branch of the target vessel, but not developed micro branch was classified as Grade-I. Both distal main branch and the micro branch of the target vessel had forward blood flow, but at least three cardiac cycles were needed to completely develop was classified as Grade-II. The target vessel micro branches were fully developed and rapidly emptied was classified as Grade-III.
- Markers of myocardial injury, including cardiac troponin T (cTnT) and creatine kinase isoenzyme MB (CK-MB) were measured by enzyme-linked immunosorbent assay. The kit was purchased from Wuhan Bodde Bioengineering Co., Ltd. CK-MB and cTnT levels were collected six ours after procedure.
- Complications, including decreased hemoglobin, gastrointestinal bleeding, hematuria, and hemoptysis. The data on complications were collected one month after the procedure.
- Major adverse cardiovascular events (MACE), including refractory angina pectoris, heart failure, MI and cardiogenic shock. The data on MACE were collected one month after the procedure.

### Statistical analysis:

All data were analyzed by SPSS25.0 software (IBM Corp, Armonk, NY, USA). The normality of the data was evaluated by Shapiro-Wilk test. The data of normal distribution were expressed as mean ± standard deviation. Independent sample t-test was used for inter group comparison, and paired t-test was used for intra group comparison. The counting data were expressed as number of cases, and Chi-square test was used for comparison. *P*<0.05 indicated statistically significant difference.

## RESULTS

A total of 156 patients were included in this study, including 79 patients in Group-I and 77 patients in Group-II. There was no significant difference in the baseline data between the two groups (P>0.05) (Table-I). Before the treatment, TIMI blood flow classification was comparable between the two groups (P>0.05). After the treatment, TIMI blood flow classification of the two groups was improved, and was significantly better in Group-I compared to Group-II (P<0.05) (Table-II). Before the treatment, there was no significant difference in serum cTnT and CK-MB levels between the two groups (*P*>0.05). After the treatment, serum concentrations of cTnT and CK-MB in both groups decreased compared to pre-treatment levels, and were significantly lower in Group-I compared to Group-II (*P*<0.05) (Table-III).There was no significant difference in the incidence of complications between Group-I (3.80%) and Group-II (6.49%) (*P*>0.05) (Table-IV).The incidence of MACE in Group-I (3.80%) was significantly lower than that in Group-II (12.99%) (*P*<0.05) (Table-V).

**Table-I:** Comparison of baseline data between the two groups.

Baseline data	Classification	Group-I (n=79)	Group-II (n=77)	χ^2^/t	P
Male (yes)	/	41 (51.90)	37 (49.35)	0.101	0.750
Age (years)	/	61.46±8.39	60.25±8.90	0.873	0.384
BMI (kg/m²)	/	24.16±3.30	24.62±3.18	-0.882	0.379
Diabetes (yes)	/	24 (30.38)	17 (20.08)	1.387	0.239
Coronary heart disease and PCI type	STEMI emergency surgery	43 (54.43)	40 (51.95)	2.004	0.572
	STEMI elective surgery	14 (17.72)	20 (25.97)		
	NSTEMI	17 (21.51)	12 (15.59)		
	UA	5 (6.33)	5 (6.49)		
Target vessel	Front drop	39 (49.37)	32 (41.56)	2.140	0.343
	Left Circumflex LCX	17 (21.52)	14 (18.18)		
	Right crown	23 (29.11)	31 (40.26)		
Number of diseased vessels	Single branch	39 (49.37)	37 (48.05)	0.699	0.705
	Double branch	33 (41.77)	30 (38.96)		
	Three branches	7 (8.86)	10 (12.99)		

***Note:*** PCI: percutaneous coronary intervention (PCI); STEMI: ST-segment elevation myocardial infarction; NSTEMI: non-STEMI; unstable angina.

**Table-II:** Comparison of TIMI blood flow classification between the two groups.

Group	Before treatment				After treatment				Z	P

	Grade-0	Grade-I	Grade-II	Grade-III	Grade-0	Grade-I	Grade-II	Grade-III		
Group-I (n=79)	31 (39.24)	29 (36.71)	17 (21.52)	2 (2.53)	0 (0.00)	1 (1.27)	1 (1.27)	77 (97.47)	-7.698	<0.001
Group-II (n=77)	33 (42.86)	26 (33.77)	17 (22.08)	1 (1.30)	2 (2.60)	3 (3.90)	7 (9.09)	65 (84.42)	-7.471	<0.001
*Z*	0.534				8.490					
*P*	0.911				0.037					

**Table-III:** Comparison of myocardial injury markers between the two groups.

Time	Group	n	cTnT (ug/L)	CK-MB (U/L)
Before treatment	Group-I	79	5.78±1.17	442.48±74.26
	Group-II	77	5.87±1.11	463.25±80.10
	*t*		-0.486	-1.680
	*P*		0.628	0.095
After treatment	Group-I	79	1.07±0.28^a^	128.41±46.04^a^
	Group-II	77	1.32±0.30^a^	189.22±58.00^a^
	*t*		-5.388	-7.263
	*P*		<0.001	<0.001

***Note:*** Compared with before treatment in the same group,

a P<0.05.

**Table-IV:** Comparison of incidence of complications between the two groups.

Group	n	Hemoglobin decline	Gastrointestinal bleeding	Hematuria	Hemoptysis	Total incidence (%)
Group-I	79	1 (1.27)	0 (0.00)	1 (1.27)	1 (1.27)	4 (5.06)
Group-II	77	2 (2.60)	2 (2.60)	0 (0.00)	1 (1.30)	5 (6.49)
*χ^2^*						0.160
*P^a^*						0.689

a , Yates’s correction.

**Table-V:** Comparison of incidence of MACE between the two groups.

Group	n	Refractory angina pectoris	Heart failure	Myocardial infarct	Cardiogenic shock	Total incidence (%)
Group-I	79	1(1.27)	0(0.00)	1(1.27)	1(1.27)	3(3.80)
Group-II	77	3(3.90)	3(3.90)	2(2.60)	2(2.60)	10(12.99)
*χ^2^*						4.311
*P*						0.038

## DISCUSSION

This study explored the effectiveness of different tirofiban administration time windows in patients with no-reflow MI during PCI. The results showed that TIMI blood flow classification in patients who received tirofiban when TIMI Grade-≥ 1 was significantly better than in patients who were administered tirofiban directly through the guiding catheter without forward blood flow. Level of myocardial injury markers, and the incidence of MACE in Group-I patients was lower than that in Group-II. Our results indicate that tirofiban can achieve certain effect in patients with no-reflow MI during PCI. However, the effect of the drug is better when administered when TIMI Grade-≥ 1 compared to the direct application of tirofiban.

Studies have showed that tirofiban is efficient for early recovery of occluded coronary artery blood flow and prevention of distal thromboembolism^13^ and the early use of tirofiban during PCI in patients with ST segment elevation myocardial infarction can make the thrombus fully dissolve and coronary blood flow remains in a good state.^14^ In agreement with our results, Zhang et al.^15^ showed that tirofiban can help improve cardiac function, reduce the levels of CK-MB, cTnT and BNP, and reduce the incidence of adverse cardiovascular events, when given directly to STEMI patients during the PCI treatment. The study also pointed out that tirofiban can block the final pathway in the process of platelet activation and aggregation, and has stronger platelet inhibitory effect, compared to clopidogrel and aspirin, oral platelet aggregation antagonists that are commonly used in interventional surgery, ischemic heart disease, angina pectoris.^15^ Guo et al.^16^ showed that the incidence of bleeding évents in patients with acute coronary syndrome treated with tirofiban on the basis of conventional dual antiplatelet therapy was significantly reduced. This further confirms the application value of tirofiban in cardiovascular disease.^14^-^16^

However, the optimal application time is still uncertain, and there are no clear recommendations in the guidelines that are used for treating patients with MI and no-reflow. Zou et al.^17^ confirmed that the TIMI blood flow classification and myocardial perfusion classification of STEMI patients who received tirofiban in the rescue and the catheter room improved significantly better compared with patients who did not get tirofiban. Additionally, tirofiban treatment was associated with more significant decrease in the level of myocardial injury markers.

A study by Zheng et al.^18^ compared the outcomes of tirofiban administration at the early and late stage of intervention, and showed that TIMI blood flow classification in the early group was better than that in the late group, and St decreased more significantly at 90 minutes after the operation. This study confirmed that tirofiban was more effective when TIMI Grade-≥ 1. We may speculate that if tirofiban is given when the blood flow of the diseased vessels is not restored, it will be difficult for the drugs to reach the diseased parts and into the microcirculation.

In contracts, when TIMI Grade-≥ 1, tirofiban can reach the target vessels and enter the microcirculation.^18^ At the same time, the half-life of tirofiban is about two hours, and the final inhibition rate of platelets is 96.0% after five minutes of treatment. Platelets start recovering 2-4 minutes after the drug withdrawal. Therefore, in this study, tirofiban was continuously pumped into the coronary artery after the initial loading dose to ensure the satisfactory blood concentration, maximize its efficacy, and ensure the treatment and good outcome of the disease.^19^,^20^

CK-MB and cTnT are highly sensitive and specific markers for clinical evaluation of myocardial injury, and their abnormal expression is positively correlated with myocardial injury.^21^,^22^ This study found that the levels of CK-MB and cTnT in the two groups were decreased after the treatment. However, serum cTnT and CK-MB levels, as well as the incidence of MACE, were significantly lower in Group-I compared to Group-II. Our results showed that tirofiban could reduce myocardial injury and the levels of cTnT and CK-MB to a certain extent, and this effect was better when TIMI Grade-≥ 1.

Our results may once again be explained by the improved ability of tirofiban, when given when TIMI Grade-≥ 1, to reach the target site, and more effectively restore local blood flow, avoid distal microvascular embolism, restore myocardial perfusion, and protect the injured and ischemic myocardium. This mode of tirofiban administration can not only prevent secondary distal embolism, and reduce thrombus load, but also restore myocardial perfusion and coronary blood flow, improve the effectiveness and safety of PCI treatment, and ensure more favorable prognosis of the disease.^23^

### Limitations:

First, it is a single center retrospective study with a small sample size and participants were not randomly assigned, which may have selection bias. Secondly, although we tried to minimize confounding factors in this study, there may be unmeasurable variables and residual confounding factors in the results. Finally, the follow-up time was short. Further higher quality research is needed to confirm our obserevations.

## CONCLUSION

Compared with the direct administration of tirofiban, tirofiban given when TIMI Grade-≥ 1 can more effectively regulate the blood flow status of target vessels, reduce myocardial injury, and reduce the risk of MACE in patients with no-reflow myocardial infarction during PCI.

### Authors’ contributions:

**CM** and **HY:** Conceived and designed the study, collected the data and performed the analysis, were involved in the writing of the manuscript and are responsible for the integrity of the study.

All authors have read and approved the final manuscript.
